# Using routine clinical and administrative data to produce information on mental health in Brazil

**DOI:** 10.11606/s1518-8787.2022056004818

**Published:** 2022-08-22

**Authors:** Flavia Jôse Oliveira Alves, Daiane Borges Machado, Jacyra Azevedo Paiva de Araujo, Luis Fernando Silva Castro-de-Araujo

**Affiliations:** I Fundação Oswaldo Cruz Instituto Gonçalo Moniz.Centro de Integração de Dados e Conhecimento para a Saúde Salvador Bahia Brasil Fundação Oswaldo Cruz. Instituto Gonçalo Moniz.Centro de Integração de Dados e Conhecimento para a Saúde. Salvador, Bahia, Brasil; II Harvard Medical School Department of Global Health and Social Medicine Boston Massachusetts EUA Harvard Medical School. Department of Global Health and Social Medicine. Boston, Massachusetts, EUA; III University of Melbourne Melbourne Victoria Australia University of Melbourne. Austin Health. Melbourne, Victoria, Australia

In Brazil, secondary health data sources are available in electronic databases of aggregated statistics and individual microdata. One of these complex databases is the *Sistema de Informação Ambulatorial* (SIA/SUS – Outpatient Information System), Datasus, which is an important source of epidemiological, administrative, and clinical information in the country. Since 1995, health records are registered via the *Autorização de Procedimentos Ambulatoriais* (APAC – Outpatient Procedures Authorization), which present a wide population coverage, providing low-cost data collection and enabling descriptive and longitudinal analyses.

Since the database available in SIA/SUS contains anonymous information on outpatient visits not the diagnosis of mental disorders for each individual, its dataset rows includes repeated information about the same individuals, which caused severe limitations to causal inference. We attempted to improve analysis through modelling, with limited results^1^. This dataset, although large, can at best describe the service use demand/supply dynamics. This is arguably fundamental information to policy-making (although mental health visits are rare in government reports), but it is much less suitable for causal inference. The SIA/SUS dataset provided us information on the use of these services by older adults^2^, as well as on visits to outpatient mental health services for children^3^ and the increased risk of postpartum psychiatric disorders due to the increase in maternal age^1^. However, it provided no information of actual possible causal links.

In 2012, the Brazilian government changed the way visits are recorded^4^. A new form was created in SIA/SUS: the *Registro das Ações Ambulatoriais de Saúde* (RAAS – Registry of Outpatient Health Actions). These changes aimed to improve details on the record of treatments in *Centros de Atenção Psicossocial* (CAPS – Psychosocial Care Centers), but hindered comparisons with data from previous years. Currently, we have accumulated experience on BigData at CIDACS/FIOCRUZ, including the creation of a new stewardship system for identified data.The model chosen by Datasus (at least in the case of mental health data) is well suited for internal auditing, but still limited for scientific use (although science is one of Datasus pillars). The challenge Datasus faces is how to release unidentified rows that can not be re-identified using the columns. This has been shown to happen in numerous cases with electronic health record data^5^. The system we think is the most effective is to anonymize individuals, removing too granular geographic information, but retaining neighbourhood level information, all the while transferring part of the responsibility for the data use to the research center interested in using data for scientific purposes by a term of responsibility and data access portals.

Finally, the availability of large regularly updated databases has been essential to the advance of clinical and epidemiological research. Despite difficulties related to the data structure, SIA/SUS presents potential for epidemiology in health services. We expect to bring attention to this important subject in order to encourage both researchers and public agents to advocate for concrete actions, as well as to help developing more effective actions in mental health care from a public health point of view.
